# Evidence-Based Alignment of Reddit Peer Advice for Depression and Anxiety: Qualitative Content Analysis

**DOI:** 10.2196/92971

**Published:** 2026-11-04

**Authors:** Yu Takizawa, Ehsan Abedin, Joanne Glover, Peivand Bastani

**Affiliations:** 1 College of Human Sciences and Culture, Flinders University, Adelaide, South Australia, Australia; 2 Institute for Mental Health and Wellbeing, Flinders University, Sturt Rd, Bedford Park, Adelaide, South Australia, 5042, Australia, 61 8 8432 4722; 3 College of Business, Creative Arts, Law and Social Sciences, Flinders University, Adelaide, South Australia, Australia; 4 Centre for Social Impact, Flinders University, Adelaide, South Australia, Australia

**Keywords:** mental health, depression, anxiety, Reddit, online forum, online communities, peer support, clinical practice guidelines

## Abstract

**Background:**

Online peer-support communities have become an increasingly important source of mental health information and support, particularly for individuals experiencing depression and anxiety. Platforms such as Reddit provide anonymous, accessible spaces where users can seek advice, share experiences, and receive community feedback. While these communities may help reduce barriers associated with stigma and access to care, concerns remain regarding the reliability, safety, and clinical appropriateness of peer-generated advice. Despite their widespread use, little empirical evidence exists regarding the extent to which highly endorsed online advice reflects evidence-based clinical recommendations.

**Objective:**

This study aimed to evaluate the extent to which highly endorsed peer advice shared on Reddit’s r/mentalhealth subreddit for depression and anxiety aligns with established national and international evidence-based clinical practice guidelines.

**Methods:**

A cross-sectional qualitative content analysis was conducted using 1065 publicly available posts from Reddit’s r/mentalhealth community related to depression and/or anxiety together with their highest-ranked responses. Posts were classified into action-based and nonaction-based advice using a large language model followed by manual verification. Action-based advice was analyzed thematically and mapped against recommendations from established, recognized national and international clinical guidelines, including the National Institute for Health and Care Excellence (NICE), the World Health Organization Mental Health Gap Action Programme (WHO mhGAP), and Australian evidence-based mental health frameworks. Thematic findings were evaluated to determine the degree of alignment with guideline recommendations.

**Results:**

Of the included posts, 327 anxiety-related and 243 depression-related discussions contained action-based advice suitable for analysis. Six overarching themes emerged: (1) seeking professional help, (2) behavioral and lifestyle changes, (3) emotional regulation and cognitive strategies, (4) interpersonal and social dynamics, (5) self-prioritization and life direction, and (6) crisis and immediate safety. Seeking professional help was the most frequently endorsed recommendation across both depression and anxiety conditions. Overall, highly endorsed advice demonstrated substantial alignment with evidence-based clinical guidance, particularly regarding professional help-seeking, behavioral activation, healthy lifestyle practices, emotional regulation, and social support. Areas of partial or limited alignment were observed primarily in discussions involving complex interpersonal circumstances, self-management beyond current evidence, and crisis situations where advice occasionally lacked the specificity or urgency recommended by clinical guidelines.

**Conclusions:**

Highly endorsed mental health advice contained within top-ranked Reddit comments generally reflects established evidence-based recommendations for managing depression and anxiety, suggesting that online peer-support forums may serve as valuable complementary sources of guidance and encouragement. Nevertheless, reliance on community-generated advice alone may be insufficient in complex or high-risk situations where professional assessment and intervention are essential. These findings provide empirical evidence supporting the potential role of moderated online peer-support communities within the broader mental health ecosystem while highlighting the importance of continued evaluation of the quality and safety of digital peer-to-peer health information.

## Introduction

People increasingly turn to digital platforms to understand and manage their health concerns, especially when they face uncertainty or limited access to professional care [1]. Online environments now function as informal “health-information ecosystems,” where individuals seek peer discussions, shared experiences, and community-generated advice. Global evidence shows rapid growth in online health-information seeking, driven by convenience, anonymity, and real-time access to user-generated content. These platforms support both symptom interpretation and emotional reassurance, making them central to contemporary health-seeking behavior [2]. However, the extent to which such peer-generated advice aligns with evidence-based recommendations remains an important public-health concern.

Mental health topics, particularly depression and anxiety, feature prominently across online forums, reflecting the high global prevalence of these conditions. Depression and anxiety are among the most common mental health conditions worldwide, affecting an estimated 5.7% and 4.7% of the global adult population [3]. Online platforms allow users to share symptoms, articulate distress, and seek reassurance from others with similar experiences. The anonymity and low barrier to participation enable open disclosure of emotional difficulties, while peer responses often blend empathy, experiential narratives, and diverse forms of lay advice [4]. Increasingly, individuals disclose psychological distress online long before seeking formal support.

Among online platforms, Reddit is the largest forum, attracting over 1 billion users worldwide in 2025 [5]. Research shows that Reddit users frequently express anxiety, loneliness, and uncertainty through highly personal narratives that incorporate slang, metaphor, and nonstandard language, making posts difficult to interpret without structured methods [6]. Evaluating how these peers’ interpretations compare with evidence-based guidance is therefore essential to prevent the spread of misinformation or misleading of the users on such a sensitive topic.

Beyond individual posts, online mental-health communities exhibit dynamic patterns of interaction. Studies mapping behavior in depression-related subreddits show that users’ emotional trajectories fluctuate in response to the tone and content of peer exchanges, rather than following a linear pathway of deterioration or recovery [7]. Such exchanges can reinforce or alleviate symptoms depending on the nature of the advice and support offered. These patterns likely extend to anxiety, where individuals rely on Reddit to interpret symptoms, trial coping strategies, and evaluate the experiences of others, highlighting the importance of determining whether community-endorsed advice reflects or diverges from evidence-based best practice.

Despite their popularity, mental health forums are not formally regulated. Anxiety disorders affect an estimated 301 million people globally [8]. Depressive disorders also account for a substantial global burden; more than 332 million people worldwide suffer from depression, which contributes significantly to disability-adjusted life years, with many individuals experiencing long delays before receiving appropriate assessment or treatment [9]. Many people first consult digital communities before engaging with mental-health professionals, relying on Reddit as an informal source of guidance [10].

Because early interpretations of symptoms strongly shape help-seeking behavior, treatment choices, and the progression of distress, assessing the accuracy and relevance of community-endorsed, evidence-based guidance is essential for public-health literacy.

Existing research on online mental health content has largely taken 2 directions. First, most computational studies use Reddit posts to identify or classify depression or anxiety-related posts, often focusing on detecting mental health states or linguistic markers rather than evaluating the appropriateness or accuracy of the advice based on evidence [11]. Second, research evaluating mental health information on websites and video platforms demonstrates variability in quality, frequent omissions of guideline-concordant recommendations, and inconsistent sourcing [12,13]. However, these studies assess static, professionally produced content rather than interactive peer-support environments, and they rarely examine anxiety-specific or depression-specific lay advice.

Online mental health advice can also be understood through theories of peer support and information behavior. Social support theory distinguishes informational support, such as advice and service recommendations, from emotional support, including empathy, reassurance, and validation. The information adoption model proposes that individuals are more likely to accept information when they perceive it as useful and credible, while social cognitive theory highlights the roles of observational learning, social reinforcement, and self-efficacy in shaping health-related behavior. Together, these perspectives offer a conceptual basis for examining not only whether peer advice aligns with clinical guidance but also how online communities may collectively generate, evaluate, and amplify forms of mental health advice [14,15].

To date, there is minimal evidence on whether the concrete self-management and help-seeking recommendations shared in Reddit’s mental-health discussions align with evidence-based guidelines. The aim of this study was to systematically compare highly endorsed Reddit advice for depression and anxiety, drawn from top-ranked comments, with evidence-based clinical guidance to identify areas of concordance and divergence between community-generated advice and guideline-based recommendations.

## Methods

### Study Design

This study used a cross-sectional qualitative content-analysis design, supplemented by quantitative descriptive summaries to characterize patterns in Reddit discussions about depression and anxiety. We analyzed publicly available posts and their top-voted comments from the r/mentalhealth subreddit to examine two key questions: (1) what kinds of advice or expressions users provide and (2) to what extent action-based guidance aligns with evidence-based recommendations.

The study proceeded in 3 stages: automated collection and filtering of relevant Reddit posts; classification of posts into action-based or nonaction-based advice; and thematic categorization and guideline comparison.

This design enabled the systematic description of online mental-health guidance at a scale while retaining interpretive depth consistent with qualitative content analysis. This study adopted a postpositivist and pragmatic orientation. Publicly available Reddit posts and comments were treated as naturally occurring text data and analyzed through a deductive and inductive direct content analysis approach.

### Data Collection

To collect data, we used Reddit’s API via the Python Reddit API Wrapper (PRAW) library. Using this script, we searched r/mentalhealth for posts containing keywords related to “depression” or “anxiety.” r/mentalhealth is a large, general-purpose peer-support subreddit open to any registered Reddit user. It is moderated by volunteer community moderators rather than mental-health professionals, and posts and comments are not clinically reviewed prior to publication. This context is relevant to interpreting both the content and community-ranking dynamics of the advice examined.

To comply with Reddit data collection policy, data were collected in weekly batches over the period from Aug 13, 2025, to Sep 14, 2025. In each weekly session, we retrieved up to 500 new posts (Reddit’s maximum for a single query) that contained the related terms in the post.

Posts were fetched and sorted by recency (newest first) to capture the latest discussions each week. This ensured coverage of ongoing conversations while controlling the volume per batch and avoiding API rate limits or data overload.

To refine the search results to posts truly relevant to depression or anxiety (and not just containing the words in passing), we used a lexicon filtering step. We defined 2 sets of indicator terms (lexicons) for each mental health topic, drawing on clinical terminology and colloquial language commonly associated with depression and anxiety [16,17]. The lexicons included terms as shown in Table 1.

**Table 1.** Lexical terms used to identify depression- and anxiety-related posts in a cross-sectional qualitative content analysis of Reddit’s r/mentalhealth community.

Depression tags	Anxiety tags
Depression	Anxiety
Major depressive disorder	Anxiety disorder
Depressive disorder	Anxious
Feeling empty	Nervousness
Depressive symptoms	Social anxiety
Sadness	Generalized anxiety disorder
Emotional depression	Panic
Hopelessness	Panic attack
Major depressive disorder	Worry
Cannot get out of bed	Overthinking
Clinical depression	Racing thoughts
Low mood	Dread
Unipolar depression	Fear spiral
No motivation	Cannot relax
Feeling numb	Constant fear
Tired all the time	Intrusive thoughts
Suicidal thoughts	Hypervigilance
Feeling empty	Angst
Nothing feels good anymore	Anxiousness
	Generalized anxiety

A post was categorized as depression-related if its text contained any one of the depression lexicon terms and similarly as anxiety-related if it contained any term from the anxiety lexicon. Many posts contained both sets of terms and were accordingly tagged in both categories (eg, a post mentioning feeling “depressed and anxious” would be labeled with both flags). Posts that did not contain any of the lexicon terms (for either category) were excluded following lexicon-based filtering.

For each post that passed the filtering process described above, we extracted the single highest-ranked visible comment at the time of data collection. Using PRAW, comments were ordered according to Reddit’s “top” sorting method, which prioritizes comments based primarily on their voting score and relative community engagement. The highest-ranked comment was then selected as the response with the greatest apparent community endorsement and prominence within the discussion.

Where the highest-ranked comment had been deleted or removed, the extraction script automatically selected the next-highest-ranked visible comment. Only 1 comment was collected per post, meaning complete comment threads and replies were not included. This produced a one-to-one pairing between each eligible post and a single representative comment. Posts with no comments, or no remaining visible comments, were excluded from the final analytic sample.

This sampling decision reflected the study’s specific aim of evaluating the advice that users were most likely to encounter when first opening a discussion, rather than conducting an exhaustive analysis of the entire comment thread. Because highly ranked comments are displayed more prominently and generally indicate stronger relative community endorsement than comments appearing lower in the discussion, they may disproportionately shape readers’ initial perceptions of what constitutes relevant, acceptable, or useful advice. Accordingly, the analysis focused on the top-ranked comment associated with each post.

Across weekly collection cycles, datasets were accumulated and subsequently aggregated at the conclusion of the data collection period to form the final analytic dataset. This dataset serves as the basis for subsequent analysis.

Table 2 provides an overview of the volume of data collected for each search term over the study period. In total, we gathered 1065 unique posts containing the target keywords, with 348 posts containing both depression- and anxiety-related keywords.

**Table 2.** Distribution of depression- and anxiety-related posts identified in a cross-sectional qualitative content analysis of 1065 posts from Reddit’s r/mentalhealth community.

Search term	Posts collected, n
Posts containing depression keywords	611
Posts containing anxiety keywords	802
Posts containing both	348
Total unique posts collected	1065

### Data Analysis

Data analysis was conducted in three sequential stages: (1) binary content categorization, (2) thematic categorization of action-based advice, and (3) assessment of alignment with evidence-based guidelines (Figure 1).

**Figure 1.**
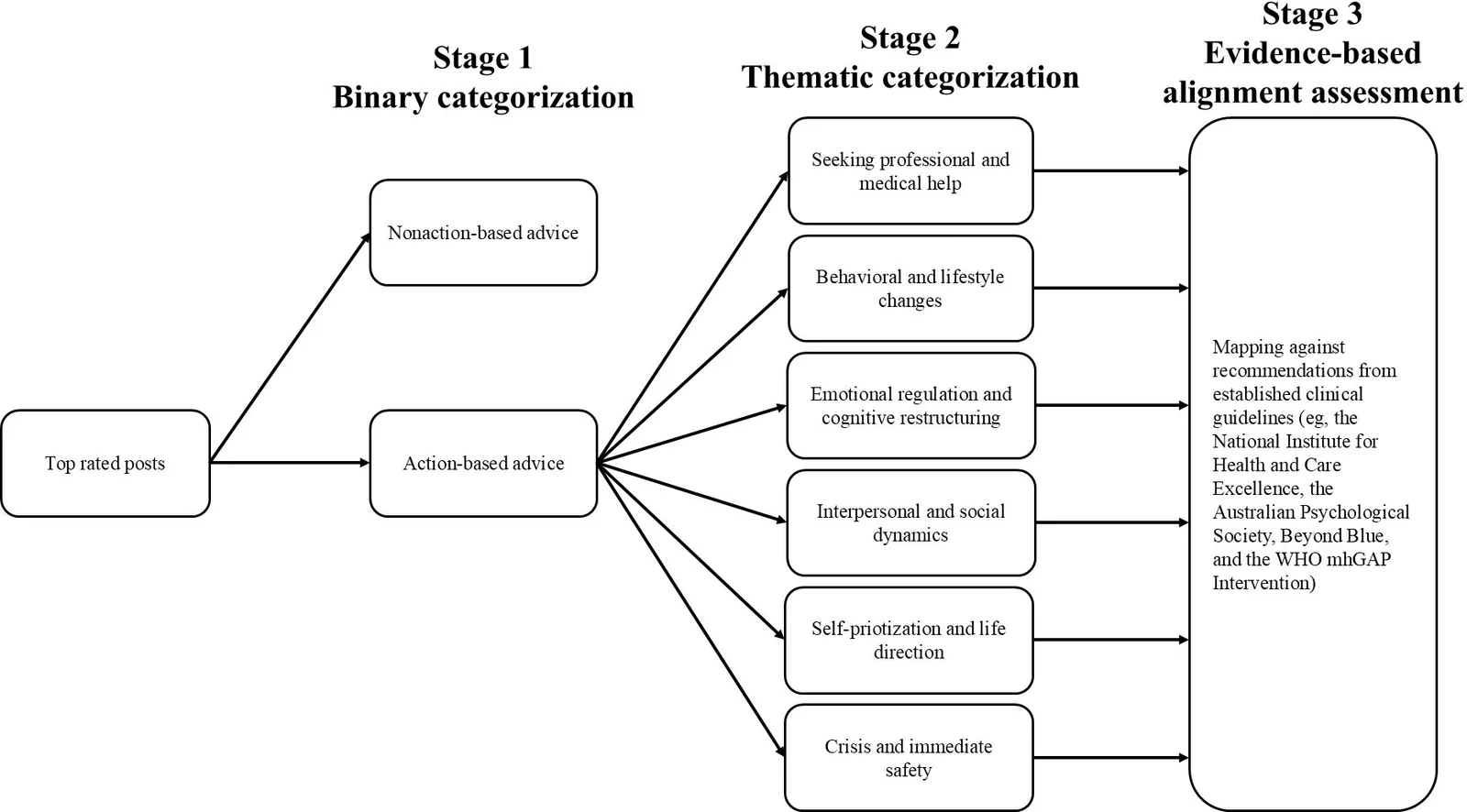
Study identification, classification, thematic analysis, and guideline-alignment process for depression- and anxiety-related posts and top-ranked comments collected from Reddit’s r/mentalhealth community. mhGAP: Mental Health Gap Action Programme.

#### Binary Classification of Reddit Posts

All collected posts were first classified into 1 of 2 categories: (1) action-based advice (posts offering concrete suggestions or directives such as recommending medication, lifestyle strategies, or coping behaviors) and (2) nonaction-based expression (posts focused on emotional sharing, empathy, or narrative reflection without proposing specific actions). Comments containing both emotional support and actionable advice were classified as action-based advice if they included at least one clear recommendation, directive, or suggested course of action. Comments that contained only empathy, reassurance, emotional validation, or personal reflection without a specific recommendation were classified as nonaction-based expressions. A subset of 25 records from the depression-related subset and 25 records from the anxiety-related subset were randomly selected and independently classified by 2 researchers (EA and PB). The researchers agreed on the classifications of all 50 records, corresponding to 100% agreement and a Cohen κ of 1.00. The 50 human-labeled records were then embedded within a structured prompt as examples of the 2 categories.

The finalized prompt instructed GPT-3.5 Turbo (OpenAI) to classify each new record as either action-based advice or nonaction-based expression [18]. It contained the category definitions, classification instructions, required output labels, and the 50 human-coded exemplars. The prompt also specified the decision rules for classification and instructed the model to return a single category label for each record. Inference was conducted with temperature set to 0 to maximize consistency and minimize variation across repeated classifications.

GPT-3.5 Turbo was applied to the full dataset to generate a preliminary classification for each record. Classifier performance was evaluated against human-consensus classifications using a separate random validation sample of 100 records. The model demonstrated high accuracy, precision, recall, *F*_1_-score, and agreement with human coding across the evaluation measures presented in Table 3.

**Table 3.** Performance of the predictive model against human-consensus classifications in a random validation sample of 100 Reddit records.

Performance measure	Value
Accuracy	0.97
Precision	0.98
Recall	0.97
*F*_1_-score	0.97
Cohen κ (agreement)	0.94

The model-generated classifications were treated as preliminary rather than final decisions. All records classified by GPT-3.5 Turbo as action-based advice were subsequently reviewed by the research team. Ambiguous, borderline, or inaccurate classifications were discussed by 2 researchers and corrected through consensus. The model therefore supported the initial screening process, while final decisions regarding inclusion in the action-based advice dataset remained under human researcher control. Following this AI-assisted classification and manual verification process, 327 anxiety-related and 243 depression-related records were retained as containing action-based advice.

#### Manual Checking and Thematic Categorization of Action-Based Advice

Posts classified as action-based advice underwent further qualitative content analysis to identify the specific types of actions being recommended. Using a multistep approach, we first selected a random sample of 100 action-based posts for inductive open coding. Two researchers (PB and YT) independently reviewed this subset and generated preliminary codes reflecting the nature of the advice (eg, medication use, lifestyle modification, therapy seeking, maladaptive coping, and peer-support strategies).

Through iterative comparison, constant comparison, and discussion, the 2 sets of preliminary codes were merged, refined, and organized into a stable set of higher-order thematic subcategories, consistent with accepted methods for developing and consolidating thematic frameworks [19]. This coding framework was then applied to the full action-based dataset. GPT-generated outputs were used only as a first-pass grouping tool, after which all assigned subcategories were manually reviewed for accuracy, coherence, and conceptual fit.

To assess the reliability of subcategory coding beyond the initial 50-post training set, a random 10% subsample of the action-based dataset (n=57) was independently double-coded by 2 researchers against the finalized 26-subcategory framework, yielding 91% agreement (Cohen κ=0.87). Discrepancies were resolved through discussion and, where necessary, consultation with a third team member. The finalized codebook was then applied to the remaining dataset by consensus-trained coders.

Frequency counts were calculated across subcategories to describe the distribution of advice types in depression-related posts, anxiety-related posts, and posts referencing both conditions. These descriptive summaries supported the interpretation of thematic patterns within the dataset.

#### Evidence-Based Alignment Assessment

To evaluate whether the action-based advice aligned with evidence-based mental-health practices, each subcategory was mapped against recommendations from established clinical guidelines, including the National Institute for Health and Care Excellence (NICE), the Australian Psychological Society (APS), Beyond Blue, and the World Health Organization Mental Health Gap Action Programme (WHO mhGAP) Intervention Guide. For each subcategory, alignment was coded as aligned, partially aligned, or nonaligned based on concordance with recommended clinical strategies for depression and anxiety.

We combined international guidelines (eg, WHO mhGAP and NICE) with Australian frameworks (eg, APS, RANZCP, and Beyond Blue) because Australian guidance is largely built on international recommendations but adds local service-pathway detail. Advice was coded as aligned when consistent with both, partially aligned when consistent with only one, and nonaligned when consistent with neither. We did not find direct contradictions between the 2 guideline sets for the subcategories examined; differences were mostly about level of detail, not conflicting advice. Guideline alignment assessments were independently reviewed by 2 members of the research team. Where disagreements or borderline cases arose, classifications were discussed and resolved through consensus, with consultation from a third team member when required. Formal interrater reliability statistics were not calculated for the guideline-alignment stage.

### Ethical Considerations

This study analyzed publicly available, anonymized user-generated content from the Reddit subreddit r/mentalhealth. Ethical approval for the project was granted by the Flinders University Human Research Ethics Committee, Australia (Project ID: 8797), which approved the use of publicly accessible online data and issued a waiver of consent. Approval was provided on August 7, 2025, and remains valid until August 7, 2030, subject to standard annual reporting. All procedures complied with the National Statement on Ethical Conduct in Human Research [20], Reddit’s Terms of Service, and conditions outlined by the Committee, with no attempts made to identify individual users.

Collected posts and comments were stored on a secure, access-controlled institutional server accessible only to the research team. Reddit usernames, profile links, and other directly identifying metadata were removed prior to analysis and are not reported in this manuscript. Where illustrative excerpts are presented in the Results (Table 4), each entry is a paraphrased summary that may include a single short (under 15-word) direct quotation from the original comment. Examples were selected to exclude posts from self-identified minors and to avoid reproducing detailed crisis narratives, to reduce the risk of reidentification.

**Table 4.** Illustrative top-ranked Reddit comments across advice categories and subcategories for depression and anxiety in a cross-sectional qualitative content analysis of r/mentalhealth discussions.

Category and subcategory	Illustrative comments
Seeking professional and medical help	
Initial contact and referral	Psychiatric nurse commenter suggested talking to a counselor about how mental health was affecting grades, noting “you might need some accommodation” such as extended test time
Diagnosis and screening	Warned that TikTok mental-health content is “often exaggerated or intended to be fun, cute, quirky,” and can lead to inaccurate self-diagnosis, recommended seeing a professional instead
Medication and treatment	Encouraged honesty with a provider about dependence, noting they “won’t cut it immediately” but will help you gradually withdraw
Specific modalities	Suggested that if behavior therapy doesn’t work, it may be worth trying to “try emotion regulation after,” while noting some controversy around therapy that pushes neurotypical standards
Seeking second options	Shared having been “misdiagnosed with Depression and Anxiety” years earlier before a later borderline personality disorder diagnosis, and encouraged the poster to keep seeking further evaluation
Behavioral and lifestyle changes	
Starting/maintaining action	Credited “forcing myself to socialize & deal with the uncomfortable feelings of anxiety” with making it easier over time
Physical health	Warned that alcohol only makes things worse and encouraged the poster to “do whatever you can to stop,” offering to share suggestions
Routine and structure	Described how they “gamify my routines and have a reward after” to make small tasks feel more engaging, starting little by little rather than forcing it
Environment and distraction	Said they do “increasing more difficult multiplication problems in my head” to quiet a racing mind at bedtime
Emotional regulation and cognitive restructuring	
Mindfulness and grounding	Suggested breathing exercises and “dropping the story” you’re creating around difficult sensations and just staying focused on breath and body instead
Emotional processing	Explained that the numb, disconnected feeling is called emotional blunting and can follow stress, burnout, or trauma—like “your brain hits the mute button” to cope
Self-talk and mindset	Reassured the poster they were not ungrateful, just struggling, and encouraged them to “try not to guilt trip yourself”
Introspection and awareness	Suggested starting a thought journal to track triggers and responses, framing the first step as needing to “build insight into yourself”
Interpersonal and social dynamics	
Setting boundaries	Speaking from clinical practice, noted that “your nervous system doesn’t care how good things look on paper,” explaining that old patterns can override circumstances even when life looks fine externally
Seeking/building support	Said that “small acts of kindness, patience, and listening” without trying to fix everything can help, along with gentle shared routines that let a partner move at his own pace
Handling toxic people	Advised to “stop enabling them unless you’re getting something in return,” noting the person has no reason to change as long as they keep getting validation
Support for others	Suggested both partners likely grew up feeling invalidated and framed the path forward simply: “validation, invalidation is how you fix it”
Self-prioritization and life direction	
Self-focus and autonomy	Reassured the poster they were not alone and said, “you can’t allow your bf’s mood to dictate how you feel,” urging them to focus on their own recovery instead
Life purpose and change	Posed a set of reflective questions, “what does make you happy?” and what could make it last, as a way to identify what might be missing
Acceptance and identity	Said relying on external validation to define yourself does not work, and it’s time to “figure out who you really are” instead
Crisis and immediate safety	
Emergency contact	Encouraged disclosure, noting that “the NHS will prioritize you more if you explain that you’re suicidal,” and that a facility placement cannot happen without consent
Physical safety	In response to a question about combining daytime hydroxyzine with nighttime trazodone, commenter advised against it, citing “the risk of messing with my heartbeat”
Seeking urgent help	Said admission was unlikely for those symptoms alone, but advised to “weigh your options, do your research” and see a professional if ever feeling like a danger to themselves or others
Nonactionable/reflective	
Shared experience/empathy	Shared that even doing everything right, medication, therapy, exercise, you can still “wake up one morning and completely relapse for no reason”
Probing/reflective question	Guessed the poster had gone along with others’ expectations growing up, “you weren't being you, you were being a part of them,” and asked if that resonated
General encouragement	Older commenter reassured the poster, “please don’t think that you’re already too old,” saying they'd once felt the same way at that age

### Researcher Characteristics and Reflexivity

The coding team brought complementary backgrounds: PB in public health and digital health systems, EA in computational text analysis and NLP, and YT in psychology, including prior clinical experience as a counseling psychologist. This mix supported both the technical side of the large language model-assisted classification and the clinical judgment needed to assess guideline alignment. None of the coders had any prior relationship with r/mentalhealth or its users, and their interaction with the platform was limited to data extraction. To reduce the influence of individual assumptions, initial coding was done independently by 2 researchers before a consensus discussion, with disagreements resolved through discussion and, where needed, by a third team member. We recognize that YT clinical training likely shaped judgments of guideline alignment more than the other coders’ nonclinical backgrounds. This is discussed further in the Limitations section.

## Results

### Distribution of Action-Based Advice Categories

The model classified 327 anxiety-related and 243 depression-related action-based Reddit posts into six overarching categories: (1) seeking professional medical help, (2) behavioral and lifestyle changes, (3) emotional regulation and cognitive restructuring, (4) interpersonal and social dynamics, (5) self-prioritization and life direction, and (6) crisis and immediate safety.

The proportional distribution of these categories for anxiety-related and depression-related posts is presented in Tables 5 and 6.

**Table 5.** Proportional distribution of the 6 action-based advice categories among 327 anxiety-related posts and their top-ranked comments collected from Reddit’s r/mentalhealth community.

Category	Percentage, %
Seeking professional and medical help	32.5
Behavioral and lifestyle changes	14.5
Emotional regulation and cognitive restructuring	25.8
Interpersonal and social dynamics	16.8
Self-prioritization and life direction	7.0
Crisis and immediate safety	2.5

**Table 6.** Proportional distribution of the 6 action-based advice categories among 243 depression-related posts and their top-ranked comments collected from Reddit’s r/mentalhealth community.

Category	Percentage, %
Seeking professional and medical help	34.0
Behavioral and lifestyle changes	17.5
Emotional regulation and cognitive restructuring	15.0
Interpersonal and social dynamics	14.5
Self-prioritization and life direction	12.5
Crisis and immediate safety	5.0

Across both conditions, seeking professional medical help emerged as the most common form of community-endorsed advice, representing 33% of anxiety posts and 34% of depression posts. The ranking of subsequent categories differed between the 2 datasets.

Among anxiety-related posts, emotional regulation and cognitive restructuring was the second most frequent category, followed by interpersonal and social dynamics. In contrast, among depression-related posts, behavioral and lifestyle changes formed the second most common category, with emotional regulation and cognitive restructuring ranking third.

Across the full dataset, thematic analysis identified 26 subcategories nested within the 6 major categories, capturing the diversity of advice types offered by Reddit users, with corresponding international and Australian clinical guidelines summarized in Table 7.

**Table 7.** Advice categories, subcategories, and mapped international and Australian clinical guidance for depression and anxiety in a cross-sectional qualitative content analysis of top-ranked comments from Reddit’s r/mentalhealth community.

Category and subcategory	Global guidelines	Australian guidelines
Seeking professional and medical help		
Initial contact and referral	WHO^a^ Comprehensive Mental Health Action Plan; OECD^b^ Integrated Mental Health and Work Policy; PAHO MHPSS^c^ Guidance	National Mental Health and Suicide Prevention Agreement; National Mental Health Workforce Strategy; Head to Health/PHN^d^ Stepped Care Model
Diagnosis and screening	WHO mhGAP^e^ Intervention Guide; WHO ICD-11^f^; NICE^g^ Depression/Anxiety Guidelines	RANZCP^h^ Clinical Practice Guidelines; AIHW^i^ Mental Health Services in Australia; National Standards for Mental Health Services (NSMHS)
Medication and treatment	WHO Model List of Essential Medicines (Psychotropics); APA^j^ Depression Guidelines	RANZCP Mood Disorder Guidelines; TGA/AMH^k^ medication safety; NSQHS^l^ Medication Safety Standard
Specific modalities	WHO Psychological Interventions Toolkit; APA/NICE CBT^m^ and DBT^n^ Guidelines	APS Evidence-Based Interventions; RANZCP Psychotherapy Standards; MindSpot/Beacon Digital CBT
Seeking second options	WHO Quality Rights Toolkit; UN CRPD^o^ (Autonomy and Consent)	Australian Charter of Healthcare Rights; NSQHS Partnering with Consumers Standard
Behavioral and lifestyle changes		
Starting/maintaining action	WHO “Doing What Matters” Self-Help Program; OECD Behavioral Insights in Health	National Preventive Health Strategy; Beyond Blue/Black Dog Institute Behavioral Activation Framework
Physical health	WHO Global Action Plan on Physical Activity; WHO Healthy Diet and Nutrition Guidance; WHO Alcohol Guidelines	Australian Dietary Guidelines; Physical Activity and Sedentary Behavior Guidelines; NHMRC^p^ Alcohol Guidelines; National Obesity Strategy
Routine and structure	WHO Mental Health Promotion in Workplaces; ILO^q^/WHO “Healthy Workplaces”	APS Self-Management Resources: PHN Stepped-Care Self-Management Plans
Environment and distraction	WHO Urban Health and Green Spaces; WHO Mental Health at Work	State/Territory Wellbeing Frameworks; eSafety Commissioner Healthy Digital Use
Emotional regulation and cognitive restructuring		
Mindfulness and grounding	WHO Stress Management (Self-Help Plus); NICE Depression Guidelines	APS^r^ Mindfulness-Based Therapies; Black Dog Institute Guided Self-Help
Emotional processing	WHO QualityRights (Trauma-Informed); APA Emotion-Focused Guidelines	RANZCP Trauma-Informed Care Statement; National Framework for Trauma-Informed Care
Self-Talk and mindset	WHO mhGAP CBT Interventions; NICE CBT Modules	RANZCP Mood/Anxiety CPGs^s^; APS CBT Guidelines
Introspection and awareness	WHO Self-Care Interventions 2022; OECD Digital Mental Health Insights	MindSpot/This Way Up Journaling Tools; PHN Digital Mental Health Guidelines
Interpersonal and social dynamics		
Setting boundaries	WHO Social Determinants of Mental Health Framework; WHO QualityRights (Boundaries, Consent)	Australian Charter of Healthcare Rights; NSMHS Person-Centered Care
Seeking/building support	WHO Guidance on Peer Support; WHO Commission on Social Connection	National Peer Workforce Development Guidelines; Recovery-Oriented Practice Framework; National Suicide Prevention Strategy
Handling toxic people	WHO Violence and Abuse Guidance; IASC MHPSS^t^ Principles	National Plan to End Violence against Women and Children; Lifeline/1800RESPECT Clinical Frameworks
Support for others	WHO Caregiver Support (mhGAP); UNICEF^u^/WHO Family Care Guidance	WHO Caregiver Support (mhGAP); UNICEF/WHO Family Care Guidance
Self-prioritization and life direction		
Self-focus and autonomy	WHO Quality Rights; UN CRPD (Autonomy and Capacity)	National Framework for Recovery-Oriented Mental Health Services; Australian Charter of Healthcare Rights
Life purpose and change	OECD Well-Being Framework; WHO Life Skills Education Framework	Productivity Commission Mental Health Inquiry; PHN Vocational Recovery Programs
Acceptance and identity	WHO Anti-Stigma Framework; UNESCO SEL^v^ Wellbeing Education	National Stigma and Discrimination Reduction Strategy; Lived-Experience Framework (NMHCCF)
Crisis and immediate safety		
Emergency contact	WHO “LIVE LIFE” Suicide Prevention Implementation Guide; IASC MHPSS Minimum Services Package	National Suicide Prevention Strategy; Life in Mind National Communications Charter; Lifeline/Beyond Blue Clinical Frameworks
Physical safety	WHO Preventing Suicide: A Resource; WHO mhGAP Emergency Guidelines	RANZCP Emergency Psychiatry Statement; NSQHS Comprehensive Care Standards
Seeking urgent help	WHO mhGAP Psychosis and Early Intervention Guidance; NICE Early Psychosis Pathways	Early Psychosis Prevention and Intervention Centre (EPPIC^w^); Headspace Clinical Practice Guidelines
Nonactionable/reflective		
Shared experience/empathy	WHO QualityRights (Empathy and Dignity); WHO Peer Support Services	National Peer Workforce Guidelines; Recovery-Oriented Practice Framework
Probing/reflective question	WHO Person-Centered Care Principles; OECD PROMs^x^ and PREMs^y^ Governance	ACSQHC^z^ Person-Centered Care Framework; NSQHS Partnering with Consumers
General encouragement	WHO Mental Health Promotion Framework; UNESCO/UNICEF Resilience Guidance	Mindframe Evidence-Based Communication Guidelines; National Communications Charter

a WHO: World Health Organization.

b OECD: Organisation for Economic Co-operation and Development.

c PAHO MHPSS: Pan American Health Organization Mental Health and Psychosocial Support.

d PHN: Primary Health Network.

e WHO mhGAP: World Health Organization Mental Health Gap Action Programme.

f *ICD-11: International Classification of Diseases, 11th Revision.*

g NICE: National Institute for Health and Care Excellence.

h RANZCP: Royal Australian and New Zealand College of Psychiatrists.

i AIHW: Australian Institute of Health and Welfare.

j APA: American Psychological Association.

k TGA: Therapeutic Goods Administration; AMH: Australian Medicines Handbook.

l NSQHS: National Safety and Quality Health Service Standards.

m CBT: cognitive behavioral therapy.

n DBT: dialectical behavioral therapy.

o UN CRPD: United Nations Convention on the Rights of Persons with Disabilities.

p NHMRC: National Health and Medical Research Council.

q ILO: International Labour Organization.

r APS: Australian Psychological Society.

s CPG: clinical practice guidelines.

t IASC MHPSS: Inter-Agency Standing Committee Guidelines on Mental Health and Psychosocial Support in Emergency Settings.

u UNICEF: United Nations Children's Fund.

v UNESCO SEL: United Nations Educational, Scientific and Cultural Organization Social and Emotional Learning.

w EPPIC: Early Psychosis Prevention and Intervention Centre.

x PROM: patient-reported outcome measure.

y PREM: patient-reported experience measure.

z ACSQHC: Australian Commission on Safety and Quality in Health Care.

### Alignment With Global and Australian Intervention Guidelines

The alignment between each action-based advice subcategory and established clinical guidelines is summarized in Table 7. Most subcategories corresponded to recommendations found across multiple global and Australian intervention frameworks, including evidence-based guidance for depression and anxiety management. This mapping illustrates the extent to which community-generated advice on Reddit reflects (or diverges from) recognized best-practice standards. To illustrate the nature of advice captured within each category, representative examples of top-ranked comments for each major category and condition are provided in Table 4.

## Discussion

### Principal Findings

This study evaluated whether community-endorsed mental health advice shared on Reddit aligns with evidence-based guidance for depression and anxiety. By analyzing 1065 posts from r/mentalhealth and their top-ranked comments, the findings indicate that a substantial proportion of action-based advice reflects recognized clinical strategies, particularly in relation to professional help-seeking, behavioral and lifestyle modifications, and emotional regulation techniques. At the same time, variability in alignment across advice categories highlights both the supportive potential and inherent limitations of informal, peer-driven mental-health guidance.

Beyond documenting guideline alignment, these findings describe patterns in the types of mental health information that become highly visible and community endorsed within an online peer-support setting. The observed prominence of professional help-seeking, behavioral strategies, emotional regulation, and social support is consistent with highly endorsed Reddit responses combining experiential knowledge, emotional reassurance, behavioral modeling, and informal guidance [21]. However, the study did not measure users’ credibility assessments, information-adoption processes, observational learning, reinforcement, or self-efficacy. The theoretical perspectives discussed below are therefore offered as possible interpretive lenses for the observed content patterns, not as mechanisms demonstrated by the findings.

These findings contribute to a broader body of work examining the quality and safety of digital mental health information provided outside formal clinical settings. While evidence from Bowie-Dabreo et al [22] indicates that many mental-health apps incorporate evidence-informed treatment components, important gaps remain in clinical validity, treatment fidelity, and safety. The authors emphasize the need for developers to consult relevant clinical guidelines and standards and to engage in ongoing critical reflection regarding treatment design and risk mitigation to improve intervention quality [22].

Similarly, a comprehensive scoping review of Reddit-based studies on depression and anxiety found that most prior research prioritized predictive classification, linguistic markers of distress, or professionally oriented monitoring and outreach applications, with limited attention to the quality or guideline alignment of peer-generated advice [11]. By contrast, the present study directly evaluates highly endorsed recommendations contained within top-ranked comments against established International and Australian guidelines, thereby addressing a gap explicitly identified in the existing literature.

Social support theory offers 1 possible interpretive lens for the predominance of guideline-consistent advice. Online peer-support communities can provide several forms of support, including informational support through practical recommendations and referrals, emotional support through empathy and reassurance, appraisal support through validation and feedback, and companionship through shared experience. In the present study, recommendations to seek professional care, adopt behavioral strategies, regulate emotions, and draw on social relationships are consistent with these overlapping support functions. Lived experience may also make such advice more relatable and acceptable by translating formal mental health principles into language grounded in everyday experience; however, perceived relatability and acceptability were not directly assessed in this study.

The findings may also be interpreted through the information adoption model [23], which proposes that information uptake is shaped by perceptions of information quality, usefulness, and source credibility. Although Reddit users may not possess formal clinical authority, the platform’s voting and ranking mechanisms provide visible signals of collective endorsement. One possible interpretation is that highly ranked comments could be perceived as more credible, useful, or socially validated than less visible responses. Reddit’s ranking system should not be understood as a guarantee of clinical accuracy. It could be hypothesized to function as an informal community-level filtering process through which advice regarded by users as constructive, relevant, or acceptable becomes more visible. Nevertheless, the present study did not measure readers’ perceptions of credibility or usefulness, information adoption, or the reasons comments received upvotes. The information adoption model therefore provides a plausible interpretation of the observed pattern that requires direct testing, rather than an explanation established by these data.

Social cognitive theory provides another possible interpretation of how guideline-consistent advice might be socially reinforced within online communities. Descriptions of coping strategies, professional help-seeking, emotional regulation, and behavioral change could function as models for possible action. Similarly, upvotes, supportive replies, visibility, and community recognition could act as forms of social reinforcement, while narratives of successful coping might support readers’ perceived self-efficacy. Contributors might also be motivated by altruism, recognition, and feedback to provide advice they regard as useful [24]. These propositions are theoretically consistent with social cognitive self-efficacy, but observational learning, reinforcement, self-efficiency, and contributor motivations were not measured. They should therefore be treated as hypotheses for future research rather than mechanisms demonstrated by the present findings. Taken together, these perspectives raise the possibility that the observed guideline alignment reflects more than individual users independently reproducing clinical information. The pattern may be interpreted as consistent with a collective process involving experiential knowledge, public mental health messages, prior encounters with health care services, and platform-based endorsement. This interpretation constitutes a theoretical proposition arising from the study rather than an empirically tested account of how particular comments became prominent. Future studies should directly examine whether perceived support, credibility, visibility, and reinforcement predict readers’ evaluations, information adoption, or behavioral responses.

Comparative analysis of anxiety-related and depression-related posts further revealed condition-specific differences in the types of advice endorsed by the community. While professional help-seeking emerged as the dominant category across both conditions, anxiety-related discussions more frequently emphasized emotional regulation and cognitive strategies, whereas depression-related discussions showed a stronger focus on behavioral activation and lifestyle changes. These patterns are consistent with the differing symptom profiles and lived experiences associated with anxiety and depressive disorders and mirror distinctions commonly reflected in clinical guidance. One possible interpretation is that the observed alignment reflects broader dissemination of mental health concepts through public-health campaigns, prior clinical encounters, or widely available self-help resources. The study did not examine where contributors acquired this knowledge, so this interpretation remains to be tested.

Mapping advice subcategories to International and Australian clinical guidelines demonstrated substantial overlap, particularly given that many Australian frameworks are closely aligned with global recommendations. This convergence supports the robustness of the observed alignment and indicates that community-endorsed advice often reflects widely accepted best-practice principles rather than idiosyncratic or locally specific norms. Nevertheless, partial or nonaligned advice was observed in more complex domains, including crisis situations, interpersonal conflict, and deeply individualized coping strategies. In these cases, advice was sometimes overly general, insufficiently contextualized, or lacking appropriate safety framing, underscoring the risks of oversimplification in informal peer-support environments [16].

The findings have several practical implications. While not all content posted on Reddit can be assumed to be reliable or clinically appropriate, the results suggest that highly ranked comments, those most visible and most likely to be encountered by users, frequently align with evidence-based recommendations and may offer meaningful support to individuals experiencing depression or anxiety. These findings suggest that highly rated comments may represent a useful area for future investigation regarding the quality of online mental health advice. From a public-health perspective, this underscores the value of understanding platform-specific information hierarchies rather than treating online mental-health content as uniformly unreliable. Sharing these insights through public-facing summaries, educational resources, and collaboration with online communities could support safer help-seeking and contribute to improved mental health and well-being in the wider community. However, further research is needed to determine whether such approaches influence help-seeking behavior or mental health outcomes.

Beyond these implications, the findings suggest a potential contribution of lived experience within online mental health support spaces. Many highly ranked comments drew on personal experience to offer empathetic, practical guidance that reinforced or contextualized evidence-based recommendations. Because the study did not assess readers’ responses or formally evaluate lived-experience roles, it cannot establish whether these contributions affected perceived credibility, support, or behavior. The observed pattern nevertheless identifies lived-experience contributions in informal mental health settings as an important area for future research, including how such contributions might be appropriately supported, integrated, and aligned with clinical guidance.

This study has several notable strengths. It used a highly efficient and scalable content-analysis approach by combining large language model–assisted classification with comprehensive manual human verification, enabling systematic analysis of a large dataset while maintaining interpretive accuracy. The focus on top-ranked comments enhances ecological validity by analyzing advice most likely to influence readers, and the use of multiple international and Australian clinical guidelines strengthens the credibility of the alignment assessment.

### Limitations

Several limitations should also be acknowledged. Data collection was limited to approximately 1 month, which may constrain the breadth of perspectives captured and limit generalizability across time or changing community dynamics. The analysis was restricted to a single subreddit and English-language content, and only one comment per post was examined, which does not capture the full diversity of viewpoints within discussion threads. In addition, the lexicon-based filtering strategy may have excluded relevant posts describing symptoms or experiences of depression and anxiety without using predefined keywords. Consequently, some relevant discussions may not have been captured in the final dataset. Reddit users are also not representative of the general population. Compared with the broader internet-using public, they skew younger and more male, and subreddit-specific norms and moderation practices shape the tone and content of the advice exchanged [25]. These characteristics may limit the generalizability of the findings to other online communities or platforms with different demographic compositions and cultures. In addition, it was not possible to determine whether individuals offering advice were qualified mental health professionals, drawing on formal training, lived experience, or a combination of both. Nonetheless, these design choices are consistent with the study’s aim of evaluating community-endorsed advice and providing a focused assessment of the most visible and relatively community-endorsed guidance.

Furthermore, reliability for the thematic subcategory coding was checked on a subsample rather than the full dataset. Applying full-dataset, multirater coding in future work would help test how much this influenced the results. In addition, although interrater reliability was assessed for the thematic subcategory coding, formal reliability statistics were not calculated for the guideline-alignment assessments. These assessments were independently reviewed by 2 researchers and disagreements were resolved through consensus. Future studies could strengthen this stage by incorporating formal reliability testing of guideline-alignment judgments.

In addition, the analysis was limited to the single highest-ranked visible comment for each post. However, the highest-ranked comment should be interpreted as the most visible and relatively community-endorsed response at the time of data collection, rather than as evidence of unanimous community consensus or the overall quality of the discussion. Accordingly, the findings should be interpreted as reflecting highly endorsed advice rather than the full spectrum of advice exchanged within Reddit discussions. Future research should examine complete comment threads and changes in comment rankings over time to better understand how competing advice, misinformation, and corrective responses develop within online mental-health discussions.

Finally, the quantitative summaries were intended to describe patterns within the qualitatively coded dataset rather than test prespecified hypotheses. Inferential comparisons across depression- and anxiety-related subsets were not conducted because these groups were not mutually exclusive; posts containing terms relating to both conditions contributed to both subsets. Future research could classify posts into mutually exclusive depression-only, anxiety-only, and co-occurring groups and use prespecified inferential analyses to examine whether advice distributions differ across these categories.

### Conclusions

In summary, this study demonstrates that highly endorsed mental health advice on Reddit frequently aligns with evidence-based clinical guidance, particularly regarding professional help-seeking and commonly recommended self-management strategies. Although limitations and risks remain, especially in complex or high-risk contexts, the findings highlight the potential value of online peer-support spaces as complementary sources of encouragement and guidance. Future research should examine longer timeframes, additional platforms, and longitudinal outcomes to better understand how online advice exposure influences help-seeking behavior and mental health trajectories. Given the demographic and cultural specificity of Reddit’s user base, future work should also extend this analysis to platforms with different demographic compositions and community cultures to establish whether the observed patterns of alignment generalize beyond Reddit.

## Abbreviations

APS: Australian Psychological Society
NICE: National Institute for Health and Care Excellence
PRAW: Python Reddit API Wrapper
WHO mhGAP: World Health Organization Mental Health Gap Action Programme
